# Estimated radiation doses to ovarian and uterine organs in breast cancer irradiation using radio‐photoluminescent glass dosimeters (RPLDs)

**DOI:** 10.1002/jmrs.445

**Published:** 2020-10-20

**Authors:** Puntiwa Oonsiri, Chulee Vannavijit, Mananchaya Wimolnoch, Sivalee Suriyapee, Kitwadee Saksornchai

**Affiliations:** ^1^ Radiation Oncology Division Radiology Department King Chulalongkorn Memorial Hospital The Thai Red Cross Society Bangkok Thailand; ^2^ Radiation Oncology Division Radiology Department Faculty of Medicine Chulalongkorn University Bangkok Thailand

**Keywords:** Left‐side breast irradiation, out‐of‐field radiation, ovarian dose, radio‐photoluminescent glass dosimeters (RPLDs)

## Abstract

**Introduction:**

The well‐being of breast cancer patients is essential, especially fertility in patients of reproductive age. The objective of this study was to estimate the radiation doses to the ovaries and uterus for different treatment techniques of breast cancer irradiation using radio‐photoluminescent glass dosimeters (RPLDs).

**Methods:**

A Farmer‐type ionisation chamber (IBA FC‐65G) and RPLDs were used to measure in‐ and out‐of‐field radiation doses in a solid water phantom. The field sizes were set to 10 × 10 cm^2^ and 8 × 17 cm^2^ with the central axis at out‐of‐field measurement distances of 30 or 50 cm. The Rando phantom’s left breast was planned using four different techniques: two tangential standard fields with and without electronic tissue compensator (E‐comp) techniques, intensity‐modulated radiation therapy (IMRT) and volumetric modulated arc therapy (VMAT). The radiation doses in the ipsilateral ovary, contralateral ovary and uterus were measured using RPLDs.

**Results:**

The percentage ratio of out of field to in field was affected by distance from the central axis to the point of measurement, in addition to the field sizes associated with collimator scatter. Advanced techniques such as IMRT and VMAT produced higher doses to the ovaries and uterus. The estimated results of the worst‐case scenario for the ipsilateral ovary, contralateral ovary and uterus were 0.84% (42 cGy), 0.62% (31 cGy) and 0.76% (38 cGy), respectively, for a 5000 cGy prescription dose.

**Conclusion:**

The lowest to highest out‐of‐field radiation doses to the ovarian and uterine organs from breast irradiation were the two tangential field techniques, VMAT and IMRT. These advanced techniques yielded higher radiation leakage, which potentially contributed to the out‐of‐field radiation dose.

## Graphical abstract

The ovarian and uterine radiation dose in breast cancer irradiation was measured by using the radio‐photoluminescent glass dosimeters (RPLDs). The estimated results of the worst‐case scenario for ipsilateral ovary, contralateral ovary and uterus were 42 cGy, 31 cGy and 38 cGy of 50 Gy prescription dose of patient’s VMAT plans, respectively.

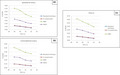

## Introduction

Seven per cent of breast cancer patients in Thailand are women of reproductive age (20–35 years).^1^ Their well‐being, especially fertility, is essential. Some patients plan to harvest eggs for in vitro fertilisation (IVF) to get pregnant once treatment is completed. The fertility threat caused by radiation is related to several factors including patient age, radiation dose, incident beam angle and the use of concurrent chemotherapy.^2^ In addition, the ovarian follicles are radiosensitive; thus, treatment may lead to atrophy with respect to a reduced primordial follicle reserve.^3^ Antypas et al.^4^ reported a small internal scattered dose of 2.1–7.6 cGy to the uterus from a 5000 cGy standard whole‐breast dose delivery with two tangential fields.

Unintentional doses outside of the treatment fields in radiotherapy arise from internal scatter within the patient, collimator scatter and leakage radiation.^5^ Advanced techniques for breast irradiation such as intensity‐modulated radiation therapy (IMRT) and volumetric modulated radiation therapy (VMAT),^6^ along with deep inspiration breath‐hold technique,^7^ attempt to avoid radiation doses to the organs at risk and increase dose coverage to the planning target volume (PTV). However, these techniques yield higher radiation leakage from the linear accelerator owing to the higher monitor unit (MU), which is similar to the prescription dose for traditional standard techniques. To our knowledge, few studies have reported on the radiation dose to the uterus and ovaries after breast irradiation.

Radio‐photoluminescence glass dosimeters (RPLDs) are a recent trend in radiation dosimetry. These passive dosimeters offer several benefits, as reported by Oonsiri et al.,^8^ including small size, high sensitivity, photon energy, dose rate independence, angular independence in the beam direction perpendicular to the longitudinal axis of the RPLD and adequate repeated reading until the detectors become annealed. These advantages make RPLDs more robust than thermoluminescence dosimeters (TLDs) for radiation dosimetry.^5^, ^9^ Unlike TLD and optically stimulated luminescence (OSL), RPLDs do not require individual sensitivity correction factors.^5^ Therefore, the present study selected RPLDs for dose measurement at the ovaries and uterus in a Rando phantom with tissue‐simulated materials.

The purpose of our study was to estimate the ovarian and uterine radiation doses in breast cancer irradiation using different treatment techniques using RPLDs. Objective data on out‐of‐field radiation levels during breast cancer irradiation are needed to scientifically support radiation oncologists determining adequate treatment for patients of reproductive age.

## Materials and Methods

### Validation of RPLDs compared with IC: solid water phantom

The performance of RPLDs concerning their sensitivity to low dose–response out‐of‐field dose measurement was validated by comparing it with that of the ionisation chamber (IC). The solid phantom dose measurement was performed in two steps; namely, by IC and RPLDs.

An IC (FC‐65G, IBA Dosimetry GmbH, Schwarzenbruck, Germany) and DOSE‐I electrometer (IBA Dosimetry GmbH, Schwarzenbruck, Germany) were used to perform the in‐field and out‐of‐field dose measurements in the solid phantom before RPLD exposure. Two stacks of slab solid water phantom measuring 30 × 30 × 25 cm^3^ (width × length × height) were set. We positioned the IC at 5 cm depth with a 10 × 10 cm^2^ field size and a source to axis distance (SAD) of 100 cm. The delivery doses were 100, 150, 200, 300, 500, 1000 and 2000 cGy from 6 MV photon beams using a Varian TrueBEAM with a Millennium 120 MLC (Varian Medical System. Inc., Palo Alto, CA, USA). For in‐field measurements, the central axis was at the centre of the chamber. For the out‐of‐field measurements, the treatment couch was moved to the gantry in the longitudinal direction for 30 cm to accurately set up the chamber at the central axis. The couch position was then returned to the previous position to provide the same dose level. The couch movement was performed to confirm that the IC position remained at the centre of the measurement point.

**Figure 1:**
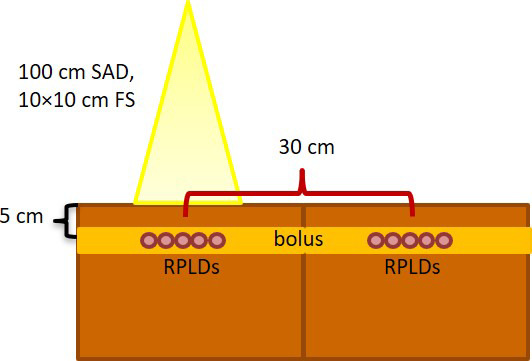
The RPLDs set up in solid water phantom both in‐field and out‐of‐field measurement.

This study utilised RPLDs (GD‐302M, batch number FD7131213‐2)^8^ for dose measurement under the same conditions as those used for the IC measurements. The RPLD process was started from the annealing process (400°C for 1 h), radiation exposure, preheat (70°C for 30 min) and signal readout using FGD‐1000 software (AGC Techno Glass Co., LTD, Shizuoka, Japan). Five RPLDs were exposed at each step dose of both in‐field and out‐of‐field irradiation, as depicted in Figure 1. The RPLDs were embedded with boluses at 1 cm superior and inferior to reduce the air gap of the solid phantom and to protect the dosimeter from cracking due to the weight of the solid phantom. A similar distance to the ion chamber measurement; namely, 30 cm between the in field and the out of field was used for the RPLDs. The effect of field size at 8 × 17 cm^2^ was also investigated in addition to 10 × 10 cm^2^. Finally, the effect of distance to out‐of‐field dose was validated at 50 cm from the isocentre in the solid phantom. Two‐tailed Student’s paired t‐tests were used to evaluate the significance of differences in signal response of RPLDs with IC at a 95% confidence level.

### Comparisons of ovarian and uterine doses using a different technique: Rando phantom

The female adult anthropomorphic Rando phantom (Alderson Research Labs, Stanford, CA) was immobilised on Vaclock (CIVCO Medical Solutions, Orange, USA) for set up reproducibility between simulation and treatment delivery. The phantom consisted of 36 transverse sections 2.5 cm in thickness. We scanned the phantom using a Siemens SOMATOM Definition AS 64‐slice CT simulator with a 3‐mm slice thickness. The scan range started from the mid‐ear of the phantom to the L2 lumbar vertebrae, which covered the whole‐breast tissue as commonly practised in our clinic. The anteroposterior and left lateral set up fields were created using Advanced Simulation MD software (GE Medical System, Waukesha, WI, USA). Both field set ups were marked on the Rando phantom via PICTOR® 3D (LAP laser, Lüneburg, Germany). This sign was used for the set up position of the Rando phantom in the treatment delivery process.

The contouring of the clinical target volume (CTV), internal mammary node (IMN) and organs at risk were completed by a single radiation oncologist. The CTVs of the breast and regional lymph nodes were contoured based on the Radiation Therapy Oncology Group (RTOG) guidelines. The CTV of the whole breast was contoured medially at the sternal rib junction, posteriorly at the pectoralis muscle excluding ribs, inferiorly at 2 cm below the breast tissue and superiorly at the inferior to the head of the clavicle. The PTV added a 5‐mm expansion from the CTV in all directions except for the skin surface. The PTV was extracted from the skin surface for 5 mm. The volume of the IMN was assessed medially at the sternum, inferiorly at the cranial border of 4th or 6th ribs for IMNs positive in imaging and posteriorly at the pleura including fat. The IMN was outlined with a 5 mm brush size with a 2‐ to 3‐mm margin to the PTV‐IMN. All organs at risk included the contralateral breast, ipsilateral and contralateral lung, heart and left anterior descending artery region were also outlined. However, the ovary and uterus were not delineated for planning.

The left breast was planned using the Eclipse treatment planning system version 15.6 (Varian Medical System. Inc., Palo Alto, CA) including the anisotropic analytical algorithm (AAA) heterogeneity calculation in four different techniques: two tangential standard fields with and without electronic tissue compensator (E‐comp) techniques, which are forward planning and deliver homogenous dose distributions to irregular surfaces via dynamic multileaf collimators (dMLC), IMRT and VMAT for 200 cGy/fraction in 25 fractions. The parameter set up for planning is shown in Table 1. The dose constraints for the IMRT and VMAT plans were *D*_95%_ = 5000 cGy (maximum dose <107% and minimum dose ≥95%) for PTV, mean heart dose ≤1000 cGy, *V*_20_ ≤ 35% of the ipsilateral lung and a mean ipsilateral lung dose ≤1800 cGy.

**Table 1:** Parameters for left breast treatment planning in a Rando phantom for different techniques.

Techniques	No. of treatment fields (Gantry angle)	Total MU
Std. fields with 30° dynamic wedge	2 (125°, 300°)	233
Std. fields with E‐comp	2 (125°, 300°)	326
IMRT	7 (100–305°, 30° separation)	2401
VMAT	3 (135–320° CCW‐CW‐CCW)	543

Abbreviations: IMRT – intensity‐modulated radiation therapy, VMAT – volumetric modulated radiation therapy, MU – monitor units, E‐comp – electronic tissue compensator, CCW – counter‐clockwise, CW – clockwise, Std. fields – standard field technique.

**Figure 2:**
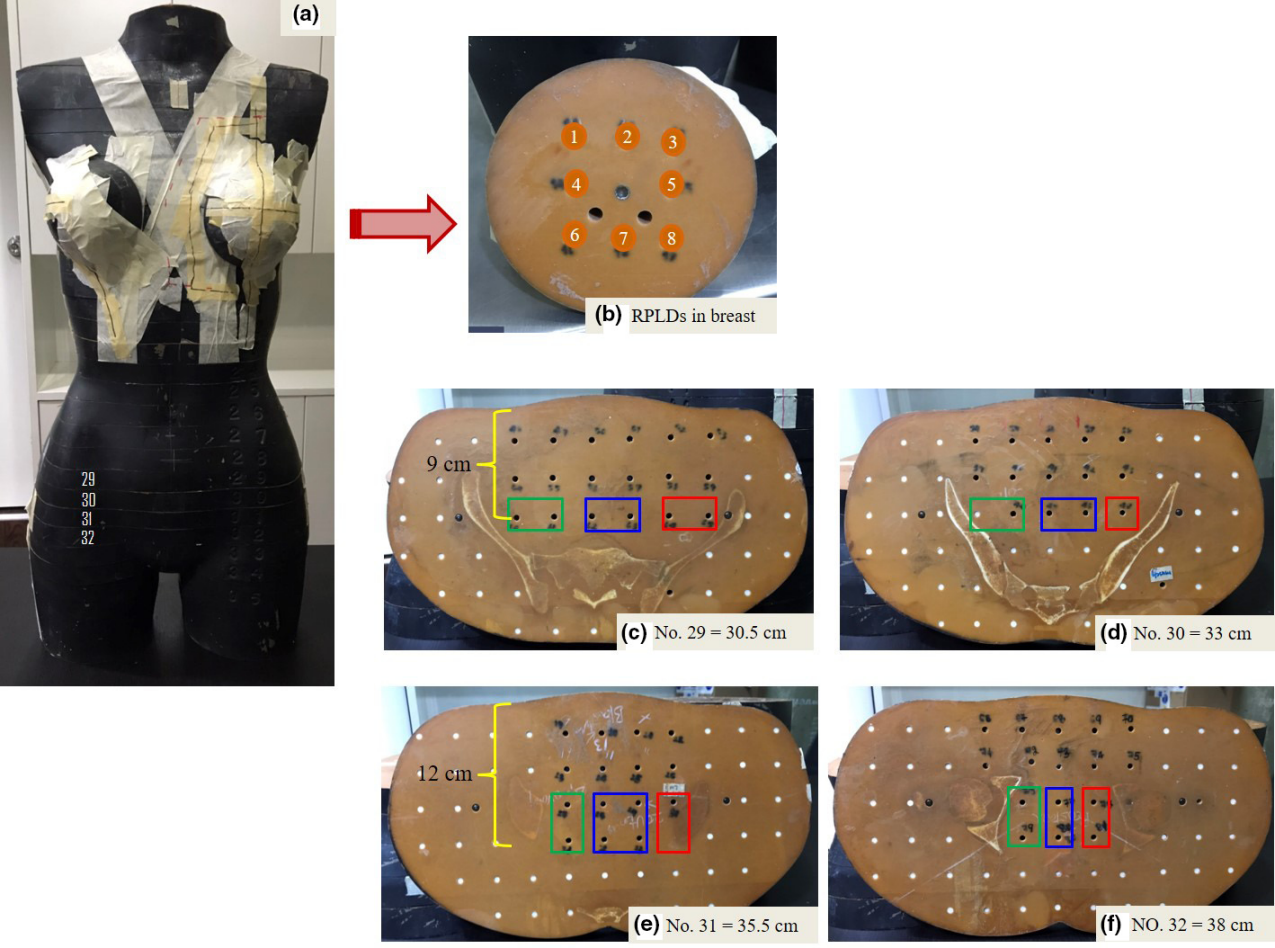
The locations of RPLDs measurement both in field and out of field: (a) Rando phantom position, (b) in the breast, (c‐f) in Rando phantom sections 29, 30, 31 and 32 located 30.5, 33, 35.5 and 38 cm inferior to the isocentre field represented ipsilateral ovary (red box), contralateral ovary (green box) and uterus (blue box).

We inserted the RPLDs into the treatment fields at the left breast and out of field, ipsilateral ovary, contralateral ovary and uterus in Rando phantom section number 29–32, according to Atlas anatomy. The Rando phantom sections were located 30.5, 33, 35.5 and 38 cm inferior to the isocentre field. The RPLDs were placed at 9 and 12 cm depths from the Rando phantom surface. The locations of the ovaries and uterus in each section are illustrated in Figure 2. Only one RPLD was placed in each measured position of the Rando phantom section. The doses were measured only once in one plan for each technique because the RPLD dose–response stability was within 2%.^8^

### Ovarian and uterine doses in VMAT plans: Rando phantom

Five VMAT patient plans of the left breast duplicated MLC movement and MU delivery into the Rando phantom. The RPLDs were used to measure the left breast, which represented the in‐field measurements. The ovarian and uterine radiation doses were also measured, which represented the out‐of‐field measurements. The start and stop gantry angles for the three partial‐arc VMAT plans were 135–320° CCW‐CC‐CCW for all plans. The cause of MU variation was the complexity of the different plans. We have observed an increasing number of breast VMAT plans in our clinic, especially for IMN involvement.

## Results

### Validation of RPLDs and IC: solid water phantom

The mean percentages of out‐of‐field to in‐field signal (% OF/IN) at 30 cm from the central axis for a 10 × 10 cm^2^ field size with the IC and RPLDs in the solid water phantom were 0.21 ± 0.00% and 0.25 ± 0.01%, respectively. The minimum detectable out‐of‐field RPLD was 289.8 µGy, which corresponded to a dose setting of 100 cGy for in‐field exposure. Dose levels below 100 cGy of in‐field exposure were excluded from this study. The % OF/IN did not differ significantly between the IC and RPLDs (*P* = 0.19). The % OF/IN values for different distances from the central axis and different field sizes assessed using an IC and RPLDs are shown in Table 2. The results confirmed that different distances and field sizes directly affected the out‐of‐field radiation doses. The out‐of‐field radiation dose decreased with long distances from the field edge. The large field size also increased the out‐of‐field radiation dose due to the higher scattered collimators and treatment beam scatter within the patient.

**Table 2:** The percentage of out of field normalised to the in‐field signal (% OF/IN) of ionisation chamber and RPLDs measurement in different field sizes and distances.

Distance (cm)	Field size (cm)	OF/IF signal ratio (%)	
		Ion chamber	RPLDs
30	10 × 10	0.21 ± 0.00	0.25 ± 0.01
	8 × 17	0.28 ± 0.00	0.34 ± 0.01
50	10 × 10	0.04 ± 0.00	0.04 ± 0.00
	8 × 17	0.06 ± 0.00	0.07 ± 0.01

RPLDs: radio‐photoluminescent glass dosimeters, OF/IF signal ratio: out‐of‐field–to‐in‐field signal ratio.

### Comparisons of ovarian and uterine doses using a different technique: Rando phantom

**Figure 3:**
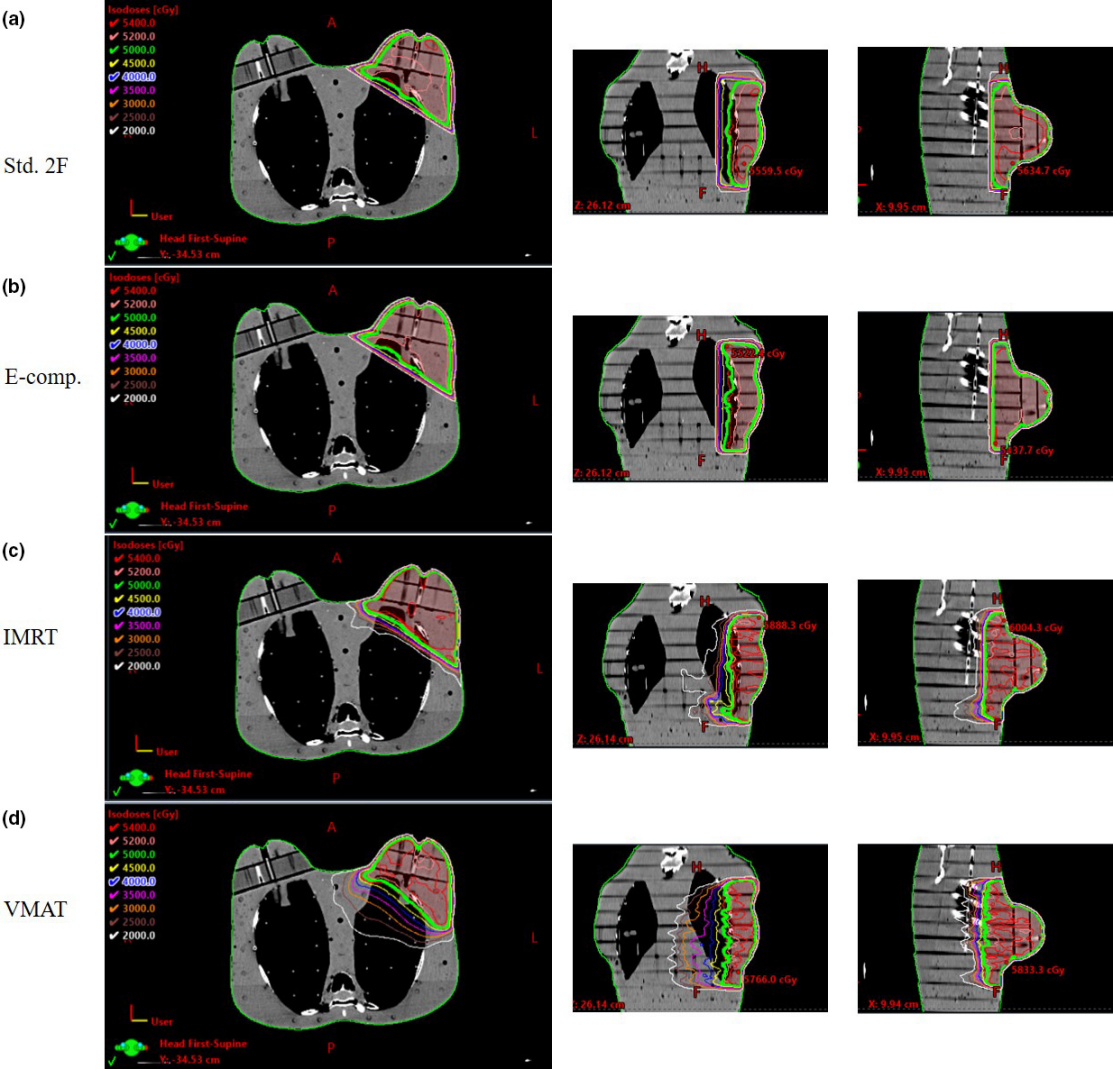
The isodose distribution for the treatment plans of each technique: (a) two tangential standard field, (b) electronic tissue compensator (E‐comp), (c) intensity‐modulated radiation therapy (IMRT) and (d) volumetric modulated radiation therapy (VMAT).

**Figure 4:**
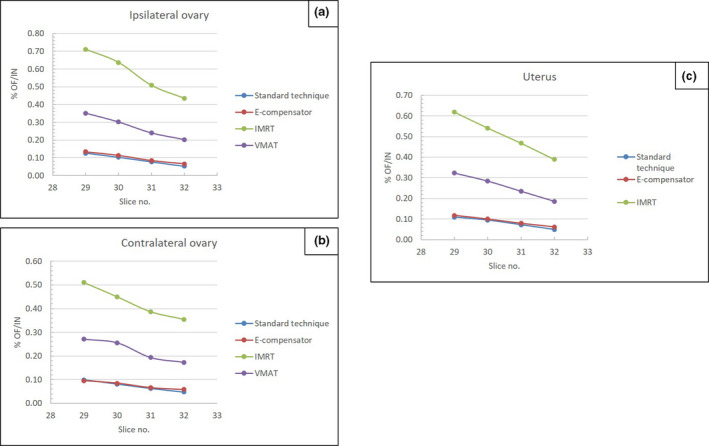
The percentage of the out of field normalised to the in‐field dose measurement (%OF/IN) of different technique for (a) ipsilateral ovary, (b) contralateral ovary and (c) uterus.

The isodose distributions for the treatment plans for each technique are illustrated in Figure 3. The PTV coverage for all techniques reached the criteria that 95% of the PTV volume was covered by 5000 cGy of the prescribed dose. The MU delivered by a different technique is shown in Table 1. The percentages of the ipsilateral ovary, contralateral ovary and uterus dose normalised to the left breast central axis are depicted in Figure 4. The % OF/IN was reported as the remaining out‐of‐field radiation dose because it can be used to estimate out‐of‐field radiation dose with different dose fractionation schedules. The contralateral ovary received a lower dose than the ipsilateral ovary it was located farther from the treatment field than the ipsilateral ovary.

The %OF/IN of the two tangential standard techniques and E‐compensator did not differ significantly owing to their shared directions and numbers of static beam entries. The %OF/IN of the standard technique and E‐compensator was lower than that of the IMRT and VMAT techniques (*P* < 0.05).

### Ovarian and uterine doses in VMAT plans: Rando phantom

The average Y direction jaw for all plans was 30.26 ± 0.98 cm. The X‐direction jaw was fixed at 16 cm because of the maximum leaf span of the MLCs in Varian linear accelerators.^10^ The mean delivery MU in the VMAT plans was 676.60 ± 33.97. The lowest to highest average %OF/IN from VMAT plans for five patients were the contralateral ovary, uterus and ipsilateral ovary. The mean %OF/IN of the contralateral ovary, uterus and ipsilateral ovary doses were 0.51 ± 0.09%, 0.58 ± 0.14% and 0.66 ± 0.15%, respectively. The section slice of the Rando phantom located closest to the field size border (section No. 29) presented the highest dose. Using the worst‐case scenario as an approximate value, the out‐of‐field radiation doses for 5000 cGy of breast irradiation were 0.84% (42 cGy) for the ipsilateral ovary, 0.62% (31 cGy) for the contralateral ovary and 0.76% (38 cGy) for the uterus.

## Discussion

The results of this study showed low out‐of‐field radiation doses for long distances from the isocentre and small field size. The out‐of‐field radiation doses to the ovarian and uterus from breast irradiation were lowest for the two tangential field standard techniques (including tissue E‐comp techniques), followed by VMAT and IMRT, in increasing order. The out‐of‐field radiation dose increased consecutively from the contralateral ovary to the uterus and ipsilateral ovary.

The %OF/IN decreased with increasing distance of the measurement point from the isocentre, consistent with the results reported by Mazonakis et al.^11^ The American Association of Physicists in Medicine Task Group 36 (AAPM TG‐36)^12^ also reported that the peripheral dose decreases exponentially with its distance from the field edge. Similarly, the field arrangement also affected the out‐of‐field radiation dose due to higher collimator scatter, as reported by Sneed,^13^ along with scatter within the patient from treatment delivery.^12^

Our measurement data in the Rando phantom showed that the out‐of‐field doses to the ovary and uterus were less than 1% of the prescribed dose. The two tangential field standard techniques (including tissue E‐comp techniques) showed MU and out‐of‐field radiation doses lower than those for VMAT and IMRT, respectively. This is because the beam modulation in IMRT and VMAT techniques generates a higher MU/dose. Higher head leakage was the main cause of this outcome. Consequently, the out‐of‐field doses were slightly higher in the advanced techniques.^14^ Furthermore, the highest %OF/IN was observed in IMRT because this technique produced approximately fourfold higher MUs compared to VMAT. The IMRT technique gave the highest 2401 MU, which produced higher radiation leakage in the out‐of‐field dose. If the number of fields or the MU was reduced, the plan was not acceptable according to the criteria mentioned previously. Thus, the IMRT was planned with seven fields. However, this dose level was less than that needed to inhibit ovarian function or cause a detrimental effect. Nevertheless, the effect of low‐dose radiation on the ovaries is limited.^15^ The principle of ‘*as low as reasonably achievable (ALARA)’* from the International Commission on Radiological Protection (ICRP) is recommended for radiation protection, which is based on the linear no‐threshold dose–response hypothesis.^16^ Hulvat et al.^17^ emphasised that pregnancy or harvesting of eggs should not occur during treatment because of the small detectable radiation dose. This might occur after treatment completion.^17^ Our results may be considered supporting data for radiation oncologists advising young patients who plan to harvest eggs for IVF. However, this is a pilot study and numerous VMAT plans should be collected for further investigation.

In the present study, advanced techniques such as IMRT and VMAT introduced higher MUs, which led to higher doses of radiation leakage to the ovaries and uterus. However, the radiation doses in these organs varied greatly for each patient depending on the distance between the lower border of the field size and the ovary or uterus placement. Regarding depth, published data^18^, ^19^ showed that the out‐of‐field dose varies very little. In this study, the RPLDs were measured at depths of 9 and 12 cm from the Rando phantom surface to represent the average variation of ovarian and uterine positions in patients.

Chambers et al.^20^, ^21^ confirmed that the ovaries are very sensitive to radiation. Ovarian function was directly related to the scattered dose to the ovaries and was inhibited at radiation doses of 250–300 cGy. Yin^22^ reported dose limits for the preservation of ovarian function in IMRT for cervical cancer of *D*_max_ < 998.5 cGy, *D*_mean_ < 532 cGy and *V*_5.5_ < 29.65%. In addition, Teh et al.^23^ and Sudour et al.^24^ reported that uterine radiation doses of less than 4 Gy did not appear to impair uterine function.

The RPLDs showed 8% of maximum X‐ray self‐attenuation when the incident beam was parallel to the long axis of the RPLDs.^8^ In this study, the correction factors for the directional dependence were negligible for the VMAT plans due to the continuation of the gantry and MLC movement. It is complicated to apply correction factors for partial doses. Meanwhile, no beam entry was directed to the long axis of the RPLDs for the two parallel opposing fields and IMRT plans.

These results were principally due to the TrueBeam Linac delivery dose to the Rando phantom, where the jaw tracking worked properly during beam‐on for the IMRT and VMAT techniques. This function may help to reduce the collimator scatter dose to the ovaries and uterus, as reported previously.^25^, ^26^, ^27^ However, the collimator scatter dose arising from different models of linear accelerators is beyond the scope of the present study.

The contribution out‐of‐field dose from a millennium 120 MLC leakage as a dosimetric leaf gap (DLG) and MLC transmission was 1.0 mm and 1.45%, respectively. The different models of MLC, such as the HD‐MLC, may reduce the out‐of‐field dose causing DLG and MLC transmission compared to the Millennium 120 MLC model. However, assessment of the out‐of‐field doses from different MLC models is beyond the scope of the present study.

## Conclusion

The results of this study demonstrated the lowest out‐of‐field radiation doses to the ovaries and uterus from breast irradiation for two tangential field standard techniques (including tissue E‐comp techniques), followed by VMAT and IMRT, in order of increasing dose. The advanced techniques yielded higher radiation leakage, which may have contributed to the out‐of‐field radiation doses. However, the doses to the ovaries and uterus were approximately fivefold lower than the threshold dose for ovarian function and, thus, might not negatively affect patients of reproductive age concerned about egg harvest after breast irradiation.

## Conflict of Interest

The authors declare that there is no conflict of interest.
